# Challenges and Best Practices in Consent to Treatment at University Hospital Wishaw

**DOI:** 10.1192/bjo.2025.10336

**Published:** 2025-06-20

**Authors:** Saba Ansari, Laura Inglis

**Affiliations:** 1 Lanarkshire, Bellshill, United Kingdom.; 2 University Hospital Wishaw, Wishaw, United Kingdom

## Abstract

**Aims:** We aimed to assess adherence to the Mental Health Act Code of Practice within University Hospital Wishaw’s inpatient psychiatry setting, focusing on the documentation of consent to treatment for patients under Compulsory Treatment Orders (CTO). Compulsory Treatment Orders (CTO) authorize the treatment of mental disorders under specific legal and ethical guidelines, requiring meticulous documentation of consent. Initial reviews highlighted poor electronic documentation standards for patients under CTOs, prompting a proposed practice change to include scanning and filing consent forms electronically.

**Methods:** An initial review was conducted in December 2022 across three inpatient wards at Wishaw General Hospital, covering 57 patient records to establish the presence and adequacy of T2 and T3 documentation. A follow-up review in January 2025 re-examined 65 records to assess improvements in electronic record-keeping and documentation practices following the implementation of the proposed changes.

**Results:** The 2024 review showed that all patients under CTOs had their T2B or T3B forms properly documented in physical and electronic formats. However, only 70% had their consent status adequately recorded in the electronic clinical notes. This marked a significant improvement in electronic record-keeping from the initial 2022 review.

**Conclusion:** The integration of scanned consent forms into electronic records has enhanced the accessibility and quality of documentation, allowing for better coordination of care across multiple units. Despite these improvements, the consistent documentation of patients’ capacity and consent status during clinical reviews remains a challenge. Ongoing education for medical staff and further reviews are recommended to ensure continuous adherence to the Mental Health Act Code of Practice and improve documentation practices.

